# Occupational Health Problems and Safety Conditions among Small and Medium-Sized Enterprises: A Cross-sectional Study in Shiraz, Iran

**DOI:** 10.5334/aogh.2438

**Published:** 2019-04-01

**Authors:** Mehdi Jahangiri, Hiva Azmon, Amin Daneshvar, Farzane Keshmiri, Hamed Khaleghi, Alireza Besharati, Somaye Daneshvar, Soheil Hassanipour, Mahdi Malakoutikhah

**Affiliations:** 1 Department of Occupational Health Engineering, Research Center for Health Sciences, School of Health, Shiraz University of Medical Sciences, Shiraz, IR; 2 Student Research Committee, Shiraz University of Medical Sciences, Shiraz, IR; 3 Gastrointestinal and Liver Diseases Research Center, Guilan University of Medical Sciences, Rasht, IR

## Abstract

**Background:**

Small and medium-sized enterprises (SMEs) include a large part of manufacturing jobs and play an important role in developing national economics and employment.

**Objectives:**

The present study aimed to investigate occupational health problems and safety conditions among SMEs in Shiraz, Iran.

**Methods:**

This cross-sectional study was carried out on 711 SMEs, including 371 small enterprises (fewer than 25 workers) and 340 medium enterprises (25–99 workers), in Shiraz, Iran. The participants were selected randomly among the workplaces under the coverage of social security insurance. The researcher-made questionnaire, which consisted of demographic characteristics, the frequency rate of occupational accidents, and exposure to workplace harmful agents, were distributed among participants.

**Findings:**

The results showed there were significantly more physical and chemical harmful agents in medium enterprises compared to small ones (P < 0.001). However, the frequency rate of accidents in small enterprises was significantly higher than in medium enterprises (P < 0.001). Also, there was no significant difference between the studied enterprises in ergonomic hazards, except for awkward posture, whose frequency rate was significantly higher in small enterprises (P < 0.05). Finally, among the reported symptoms, the prevalence of eye, skin, ear, and respiratory symptoms was significantly higher in medium enterprises compared to small enterprises (P < 0.05).

**Conclusions:**

Occupational health and safety (OHS) regulations in medium enterprises have led to improved OHS conditions compared to small enterprises. Therefore, small enterprises should be included in OHS regulations.

## Introduction

Small and medium-sized enterprises (SMEs) are independent firms that employ less than a given number of workers [1]. There is no comprehensive definition and standard classification for SMEs [2]. In different countries and organizations, various classifications are found based on the number of workers. For example, the International Labor Organization (ILO), South Korea [3], and Japan [4,5] consider enterprises with fewer than 50 workers as small and those with more than 50 workers as medium [3,6]. European Commission has divided enterprises into three categories: micro (fewer than 10 workers), small (fewer than 50 workers), and medium (fewer than 250 workers) [7]. In the UK, enterprises with fewer than 250 workers are also classified as SMEs [8]. The Statistical Centre of Iran has also divided enterprises based on the number of workers so that micro enterprises are those with fewer than 9 workers, small enterprises have 10–49 workers, and medium enterprises have 50–99 workers [9].

Despite the low number of workers in SMEs, they make up a large part of manufacturing jobs and play an important role in developing national economics and employment. According to the Organization for Economic Cooperation and Development (OECD), SMEs account for about 90% of employment [10,11]. Also, according to the ILO, SMEs comprise the majority of the labor force (40% in developed countries and over 60% in developing countries) [10]. However, occupational health and safety (OHS) conditions among workers in SMEs are inappropriate for many reasons. The most important causes of poor OHS conditions are young, low-educated or illiterate workers, and a lack of or insufficient training about the occupation and its hazards [12,13]; using non-standard tools with inappropriate designs [14]; and poor lighting, high noise levels, lack of ventilation, inappropriate personal protective equipment, and limited working spaces [15,16].

Some studies have been conducted in different countries on unfavorable conditions in SMEs. For example, Japanese national studies demonstrated 72% of total occupational injuries resulting in more than four days absence from work occurred in small enterprises [5]. In addition, the study carried out by Park et al. on 5080 enterprises in Korea showed that the rates of occupational deaths in small enterprises are higher than the national rates [3]. The study conducted by Wei et al. in Shanghai, China, revealed only 23.9% of the workers in small enterprises are trained about OHS and only 12% of employers provide them with necessary personal protective equipment [17].

In Iran, there are more than 14 million workers in more than 5 million SMEs, including 750,000 manufacturing workshops under the coverage of 3,500 trade unions [18]. According to the Statistical Center of Iran, small enterprises account for more than 98% of industries and workers and more than 80% of the workforce in the country [9]. Although in recent years more attention has been paid to OHS issues in small enterprises, many of them still do not have sufficient access to OHS services, and their OHS status is not desirable [19,20,21].

Despite multiple health problems in SMEs, Iranian labor law for workplaces with at least 25 workers only requires a council of technical protection and work health to prepare the equipment and resources needed for workers’ safety and health, train staff, carry out annual occupational medical examinations, monitor and measure environmental agents in workplaces, and address OHS principles [22,23]. Therefore, OHS requirements are not mandatory in workshops with fewer than 25 workers, and it is expected that OHS conditions are not satisfactory in small-sized enterprises compared to medium and large enterprises [18,20]. The present study aimed to investigate occupational health problems (OHPs) and safety conditions among SMEs in Shiraz, Iran.

## Methods

This cross-sectional study was carried out on 711 SMEs, including 371 small enterprises (fewer than 25 workers) and 340 medium enterprises (25–99 workers) in Shiraz, the capital of Fars province in the south of Iran. The subjects were selected randomly among the workplaces under the coverage of social security insurance. A team, specially trained for the study, collected the demographic data, as well as the information on OHS problems, through individual interviews and a researcher-made questionnaire.

## Measures

The researcher-made questionnaire consisted of the following series of questions: demographic characteristics (age, work experience, marital status, educational level, occupational medical examinations, and accident experience); the frequency rate of occupational accidents and OHPs, as well as affected body parts, including eyes, skin, ears, mouth, face, head, neck, legs, lungs, heart, stomach, and kidney, in addition to mental and musculoskeletal disorders; and exposure to workplace harmful agents, including physical agents (sound, vibration, heat stress, radiation, and lighting), chemical agents (fumes, dust, and gases), and ergonomic hazards (carrying heavy loads and awkward postures).

Data were collected from October 2014 to September 2015. The data were analyzed using SPSS 16.0. Chi-square and T-test were used for statistical analysis, and the significance level of P < 0.05 was selected. The study was approved by the Ethics Committee of Shiraz University of Medical Sciences. All ethical considerations were addressed, including maintaining promises of confidentiality and obtaining informed consent from the study participants. All participants were informed about the aim of the study and were told that they could withdraw from the study whenever they wished, and written consent was obtained.

## Results

Table 1 shows the demographic characteristics of the workers. The mean age and work experience of people working for small enterprises were significantly more than those working for medium enterprises (P < 0.001). The people working for medium enterprises had higher levels of education, occupational medical examinations, and work shifts compared with employees in small enterprises (P < 0.001). However, the frequency rate of accidents in small enterprises was significantly higher than medium enterprises (P < 0.001).

**Table 1:** Demographic characteristics of the studied workers in small (n = 371) and medium (n = 340) enterprises.

Variable	Small (1–24 employees)	Medium (25–99 employees)	P-value*	Total

**Age (year)**				
Mean (SD)	52.5 (16.6)	47.5 (15.2)	< 0.001**	50.1 (15.2)
(Max-Min)	(58–16)	(56–21)		(58–16)

**Work experience (year)**				
Mean (SD)	21.7 (17.05)	14.2 (11.85)	< 0.001**	17.95 (14.45)
(Max-Min)	(37–1)	(30–1)		(37–1)

**Marital Status** No. (percent)				
Single	84 (22.64)	81 (23.8)	0.709	165 (23.21)
Married	287 (77.36)	259 (76.17)		546 (76.79)

**Education** No. (percent)				
Middle School (9 years of education)	200 (53.91)	110 (32.35)		310 (43.60)
Diploma (12 years of education)	171 (46.09)	230 (67.65)	< 0.001**	401 (56.40)

**Shift-worker** No. (percent)	10 (2.69)	165 (48.53)	< 0.001**	175 (24.61)
**Occupational medical examinations** No. (percent)	28 (7.53)	177 (52.06)	< 0.001**	205 (28.83)
**Accident experience** No. (percent)	224 (60.37)	176 (51.76)	0.020**	400 (56.26)

* T test and Chi-square test. ** p < 0.05.

**Figure 1:**
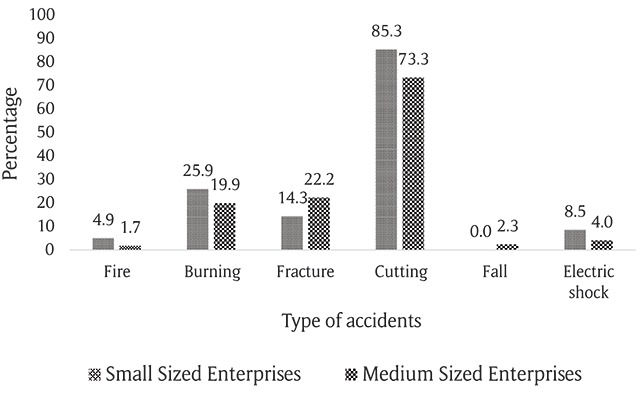
Comparison of the percentage of accident types.

Figure 1 shows the percentage of reported accidents in the studied enterprises. Accidents rates were higher in small enterprises, except for fractures and falls. This difference was significant in accident rates for electric shock, cutting, and burning (P < 0.05).

**Figure 2:**
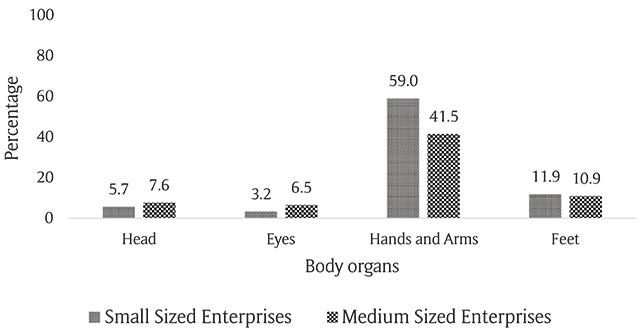
Comparison of the percentage of accidents in different parts of the body.

Figure 2 shows the percentage of accidents by injured body part among the studied enterprises. Arms and hands were the most affected regions, and the percentage of accidents were significantly higher in small enterprises (P < 0.001). The incidence rate of eye-related injuries was significantly higher in medium enterprises, and no significant difference was observed between SMEs in other occupational accidents.

Table 2 presents the frequency of harmful occupational agents in SMEs. There were significantly more physical and chemical harmful agents in medium enterprises compared to small ones (P < 0.001). There was no significant difference between the studied enterprises in ergonomic hazards, except for awkward posture, where the frequency rate was significantly higher in small enterprises (P < 0.05).

**Table 2:** Frequency of self-reported uncontrolled harmful agents among studied small (n = 371) and medium (n = 340) enterprises.

Harmful Agents	No. (Percent)		P-value*	Total No. (Percent)

	Small (1–24 employees)	Medium (25–99 employees)		

**Physical agents:**				
Noise and vibration	237 (63.88)	282 (82.94)	<0.001**	519 (73)
Insufficient lighting	61 (16.44)	92 (27.05)	<0.001**	153 (21.52)
Radiation	44 (11.76)	72 (21.17)	<0.001**	116 (16.31)
Heat stress	113 (30.46)	181 (53.23)	<0.001**	294 (41.35)

**Chemical agents:**				
Liquid chemical	196 (52.83)	180 (52.94)	1.106	376 (52.88)
Solid chemical	178 (47.98)	205 (60.29)	<0.001**	383 (53.86)
Fume and smoke	123 (33.15)	176 (51.76)	<0.001**	299 (42.05)
Chemical vapors	115 (31)	205 (60.11)	<0.001**	320 (45)
Particles	208 (56.06)	269 (79.11)	<0.001**	477 (67.08)

**Ergonomics:**				
Excessive force	292 (78.7)	264 (77.64)	0.732	556 (78.19)
Awkward posture	308 (83)	258 (75.88)	0.018**	566 (79.60)
Prolonged standing	340 (91.64)	304 (89.41)	0.308	644 (90.57)
Manual material handling (MMH)	41 (11.05)	40 (11.76)	0.764	81 (11.39)

* Chi-square. ** p < 0.05.

Table 3 shows the prevalence of OHPs reported by the workers. Among the reported symptoms, the prevalence of eye, skin, ear, and respiratory symptoms were significantly higher in medium enterprises compared to small enterprises (P < 0.05).

**Table 3:** Self-reported occupational health problems among studied small (n = 371) and medium (n = 340) enterprises.

Occupational Symptoms	No. (Percent)		P-value*	Total No. (Percent)

	Small (1–24 employees)	Medium (25–99 employees)		

Eye problems	195 (52.56)	208 (61.18)	0.020**	403 (56.68)
Skin irritation	184 (49.60)	196 (57.65)	0.031**	380 (53.44)
Hearing problems	121 (32.61)	149 (43.82)	0.002**	270 (37.97)
Headache	155 (41.78)	123 (36.18)	0.126	278 (39.09)
Respiratory symptoms	117 (31.54)	156 (45.88)	<0.001**	273 (38.39)
Mental disorders	228 (61.46)	211 (62.06)	0.870	439 (61.74)
Musculoskeletal disorders	269 (72.50)	249 (73.23)	0.827	518 (72.85)

* Chi-square. ** p < 0.05.

## Discussion

The main purpose of this study was to investigate occupational health problems and safety conditions among SMEs in Shiraz, Iran. The results showed that workers in small enterprises reported higher accident rates (224, 60.37%), less exposure to harmful agents (28, 7.53%), and a lower percentage of OHPs. However, workers in medium enterprises reported more exposure to harmful agents and a higher prevalence of OHPs.

According to the results of this study, the prevalence of occupational accidents in small enterprises (224, 60.37%) was significantly higher than in medium enterprises (176, 51.76%) (P < 0.05). This difference can be attributed to various factors, including the labor law not requiring small enterprises to form a council of technical protection and work health and to hire OHS experts. Additionally, workers in these enterprises are not usually provided with adequate safety training [4], and their levels of education are lower compared with workers in medium enterprises (Table 1). More importantly, small enterprises are less likely to be inspected by law-enforcement bodies than medium-sized enterprises. This finding is consistent with the results of Rongo et al., who investigated the OHS conditions of small enterprises in Tanzania [1]. According to Park’s study in Korea, the incidence rate of occupational accidents in small enterprises is higher than that in medium enterprises due to a lack of a good inspection system for small enterprises [3].

In this study, burns and cuts, especially on arms and hands, were the most common accidents (Figure 1), and their incidence rates were significantly higher in small enterprises compared with medium enterprises. This finding is consistent with some of the studies conducted on small enterprises, including Ghahramani, et al. [24], Jahangiri et al. [20], and Taheri [18], in Iran and Kebede Faris et al. in Ethiopia [2]. The most important reasons for the high incidence of burns and cuts in small enterprises are a lack of usage of personal protective equipment, the nature of the work (i.e., manual handling of sharp objects), the use of old machinery without protective shields, employee refusal to comply with safety rules, and a lack of adequate safety training [25].

In our study, OHPs were more prevalent among workers in medium enterprises (Table 3). This is consistent with the results investigating workplace exposure to chemical and physical harmful agents, which indicated that workers in medium enterprises were more exposed than those working for small enterprises (Table 2). The higher prevalence of OHPs in medium enterprises can be attributed to the fact that, unlike small enterprises, workers in medium enterprises are provided with occupational medical examinations and are more aware of their health status. In other words, in medium enterprises, medical surveillance is available to all workers due to mandatory medical examinations and the presence of a healthcare professional [26]. While workers in medium enterprises are aware of their health status, workers in small enterprises may have health problems they are not aware of, such as hearing impairment. Moreover, the higher prevalence of OHPs and the greater frequency of physical and chemical harmful agents in medium enterprises can be attributed to their complexity and vastness compared with small enterprises [2,3].

Small enterprises did not differ significantly from medium enterprises in terms of ergonomically harmful agents, except for awkward posture (Table 2). This is consistent with the results of Taheri [18], Jahangir [21], and Qutubuddin et al. [27]. This can be explained by the fact that new and advanced tools that address ergonomics are used in medium enterprises, while in small enterprises, ergonomic conditions of workstations are unfavorable. In addition, in small enterprises, such as automobile repair, the metal industry, the wood industry, and the chemical industry, most tasks are handled manually. Hence, inappropriately adjusted workstations, as well as non-ergonomic office desks, lead to working in awkward postures [27].

## Limitations

The most significant limitation in our study is that the data was self-reported and gathered by interview; therefore, recall bias is possible in reporting accidents and OHPs.

## Conclusion

Our study showed small enterprises reported more occupational accidents compared with medium enterprises; whereas, most OHPs were reported to be higher in medium enterprises. This can be attributed to the provision of more OHS services in medium enterprises. It can be concluded that the OHS regulations in medium enterprises have led to improved OHS compared to small enterprises. Therefore, we recommend the labor law be expanded to include OHS regulations in small enterprises.
